# Assessing ecological correlates of marine bird declines to inform marine conservation

**DOI:** 10.1111/cobi.12378

**Published:** 2014-09-05

**Authors:** L Ignacio Vilchis, Christine K Johnson, Joseph R Evenson, Scott F Pearson, Karen L Barry, Peter Davidson, Martin G Raphael, Joseph K Gaydos

**Affiliations:** * Wildlife Health Center, School of Veterinary Medicine, University of California DavisDavis, CA, 95616, U.S.A.; † Wildlife Research Division, Washington Department of Fish and WildlifeOlympia, WA, 98501, U.S.A.; ‡ Bird Studies Canada, Pacific Wildlife Research Centre5421 Robertson Road, Delta, British Columbia, V4K 3N2, Canada; § USDA Forest Service, Pacific Northwest Research Station3625 93rd Ave. SW, Olympia, WA, 98512, U.S.A.

**Keywords:** community ecology, epidemiology, forage fish, marine birds, pursuit divers, risk analysis, análisis de riesgo, aves marinas, ecología de comunidades, epidemiología, peces forrajeros, pescadores de persecución

## Abstract

Identifying drivers of ecosystem change in large marine ecosystems is central for their effective management and conservation. This is a sizable challenge, particularly in ecosystems transcending international borders, where monitoring and conservation of long-range migratory species and their habitats are logistically and financially problematic. Here, using tools borrowed from epidemiology, we elucidated common drivers underlying species declines within a marine ecosystem, much in the way epidemiological analyses evaluate risk factors for negative health outcomes to better inform decisions. Thus, we identified ecological traits and dietary specializations associated with species declines in a community of marine predators that could be reflective of ecosystem change. To do so, we integrated count data from winter surveys collected in long-term marine bird monitoring programs conducted throughout the Salish Sea—a transboundary large marine ecosystem in North America's Pacific Northwest. We found that decadal declines in winter counts were most prevalent among pursuit divers such as alcids (Alcidae) and grebes (Podicipedidae) that have specialized diets based on forage fish, and that wide-ranging species without local breeding colonies were more prone to these declines. Although a combination of factors is most likely driving declines of diving forage fish specialists, we propose that changes in the availability of low-trophic prey may be forcing wintering range shifts of diving birds in the Salish Sea. Such a synthesis of long-term trends in a marine predator community not only provides unique insights into the types of species that are at risk of extirpation and why, but may also inform proactive conservation measures to counteract threats—information that is paramount for species-specific and ecosystem-wide conservation.

Evaluación de las Correlaciones Ecológicas de las Declinaciones de Aves Marinas para Informar a la Conservación Marina

**Resumen:**

La identificación de los conductores del cambio ambiental en los grandes ecosistemas marinos es esencial para su conservación y manejo efectivo. Esto es un reto bastante grande, particularmente en los ecosistemas que trascienden fronteras internacionales, cuando el monitoreo y la conservación de especies migratorias de amplio rango y sus hábitats son logística y financieramente problemáticos. En este caso, usando herramientas tomadas de la epidemiología, elucidamos conductores comunes subyacentes en la declinación de especies dentro de un ecosistema marino, muy similar a cómo los análisis epidemiológicos evalúan los factores de riesgo para los resultados de salud negativos e informar mejor sus decisiones. Con esto, identificamos los rasgos ecológicos y las especializaciones de dieta asociados con la declinación de especies en una comunidad de depredadores marinos que podría ser un reflejo de cambios ambientales. Para lograr esto, integramos datos de conteo de programas de censos de invierno recolectados a lo largo de monitoreos a largo plazo de aves marinas llevados a cabo en el mar Salish – un gran ecosistema marino que trasciende fronteras en el noroeste del Océano Pacífico. Encontramos que las declinaciones por década en los conteos de invierno fueron más prevalentes entre los pescadores de persecución, como los álcidos (Alcidae) y los zambullidores (Podicipedidae), que tienen dietas especializadas basadas en peces forrajeros y que las especies con distribución amplia y sin colonias reproductivas locales estaban más predispuestas a estas declinaciones. Mientras que una combinación de factores posiblemente esté causando las declinaciones de especialistas de peces forrajeros, proponemos que los cambios en la disponibilidad de presas de niveles tróficos bajos pueden estar forzando cambios en la extensión invernal de aves pescadoras en el mar Salish. Dicha síntesis de tendencias a largo plazo en una comunidad de depredadores marinos no sólo proporciona percepciones únicas de este tipo de especies que están en riesgo de ser extirpadas y el por qué de esto, sino también puede informar a las medidas de conservación proactivas para contrarrestar amenazas – información que es primordial para la conservación específica de especies y del ecosistema en su totalidad.

## Introduction

Marine ecosystems worldwide face an increasing rate of local extinctions (Jackson et al. 2001), yet identifying the mechanisms driving declines in biodiversity continues to challenge ecologists. In large ecosystems, for example, limitations of scale and access can hinder conservation and monitoring of long-range migratory species, especially in ecosystems transcending international borders. As a result, identifying mechanisms driving multiple species declines in transboundary and large marine ecosystems has been particularly problematic. Pooling multijurisdictional monitoring programs to assess ecosystem-wide trends in biodiversity and abundance of entire communities could reveal important clues about the commonalities of species that are more likely to decline or stop frequenting an ecosystem. In this way, unfavorable outcomes among members of a community can be related to species’ ecological traits and dietary specializations (Lips et al. 2003; Johnson et al. 2009), which is analogous to the identification of risk factors associated with negative health outcomes in human epidemiological studies. Assessing extinction risks in declining species is not new (e.g., Pimm et al. 1988; Purvis et al. 2000), but combining practices across the fields of ecology and epidemiology through data collected at decadal time scales could reveal ecological traits and ecosystem changes that place species at risk of undergoing population declines, and thus better inform conservation.

Since the mid 1970s, fewer marine birds have Been overwintering in the Salish Sea—an important staging area for numerous marine bird species wintering in the North American portion of the Pacific Flyway (Anderson et al. 2009; Bower 2009; Crewe et al. 2012). Discerning the particular species that are frequenting this ecosystem less and what these species have in common could offer unique insights into drivers of ecosystem change in the Salish Sea. This is because most marine birds are long-lived, migratory, and at upper levels of food webs and therefore ideal indicators of changing productivity and ecosystem structure across broad spatial and temporal scales. In the California Current, for example, Hyrenbach and Veit (2003) found that seabird species assemblages shifted in response to a 10-year decrease in productivity from being dominated by cold-water species that dive in pursuit of their prey to warm-water species that predominantly feed at the surface. And in the eastern tropical Pacific, Ballance et al. (1997) demonstrated how the seabird community is structured along a longitudinal gradient in productivity reflecting prey abundance. Both these examples demonstrate how marine bird communities respond to changing environmental conditions, particularly fluctuations in abundance of prey of low trophic level, and that investigating ecosystem-level drivers of species abundance and distribution is most revealing when multiple species and broad spatial and temporal scales are examined.

In the Salish Sea, however, a consensus on the types of species being lost is lacking, and hypotheses for the mechanisms driving ecosystem-wide declines have not been tested. This is mainly because of logistical constraints and differing survey methods used in marine bird censuses conducted by multiple wildlife agencies in a transboundary ecosystem. As a result, ecosystem-wide appraisals of long-term wintering marine bird abundance trends have yet to be attempted.

**Figure 1:**
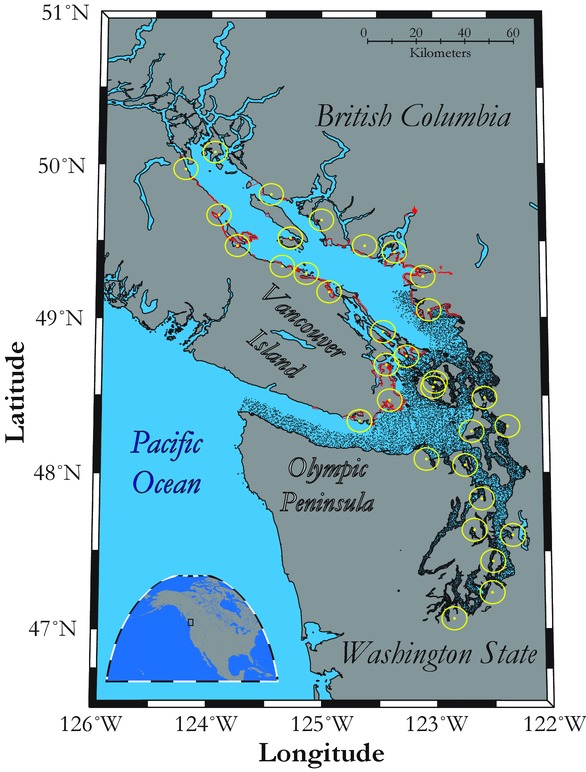
Spatial and temporal coverage of winter marine bird surveys in the Salish Sea (black dots, midpoints of 2.5 km segments from continuous aerial 100 m strip transects completed during winter months from 1994 to 2010 by the Washington Department of Fish & Wildlife [total sampling units 37,875]; red polygons, 242 survey polygons of British Columbia Coastal Waterbird Surveys completed during winter months from 1999 to 2010 [5,572 sampling units]; yellow circles, 32, 24.14-km diameter circular areas where counts were conducted during 1, 24-hour midnight-to-midnight calendar day on or around 24 December for annual Audubon Christmas Bird Counts from 1994 to 2010 [437 sampling units]).

We conducted an ecosystem-wide assessment of long-term trends in winter counts of birds in the Salish Sea—a 17,000 km^2^ marine ecosystem on North America's west coast located in Washington State in the United States and British Columbia in Canada (Fig.1). During the last 2 decades, state and provincial wildlife agencies from the United States and Canada and citizen science groups have actively monitored wintering marine bird abundances throughout this region. We analyzed these longitudinal data sets and interpreted wintering marine bird trends using an epidemiological framework—relating the incidence and distribution of unfavorable outcomes (i.e., species with regional declines past a meaningful threshold)—to determine ecological correlates that would make species less likely to overwinter in the Salish Sea. Such a synthesis required a 3-step approach: analysis of temporal trends in winter counts for all species of the Salish Sea marine bird community in all their habitats, creation of a binary variable indicating unfavorable outcomes, and use of logistic regression to assess what ecological traits increased the likelihood of species being associated with declines. This process, and other complementary analyses, helped us assess how species composition of wintering marine birds has changed in the Salish Sea. Specifically, we investigated 2 potentially complementary hypotheses that explain seabird declines in other ecosystems: declines in marine bird biomass are linked to changes in the availability of their low-tropic-level prey (Cury et al. 2011; Smith et al. 2011) and species-specific energetic costs during foraging bouts determine the type of species assemblages most likely to respond to changes in prey availability (Ballance et al. 1997; Hyrenbach & Veit 2003; Ainley & Hyrenbach 2010). We sought to provide insights into ecosystem-level drivers of multispecies declines in a community of marine predators wintering in the Salish Sea because these could be reflective of broad-scale ecosystem change. This knowledge would allow for more effective species-specific and ecosystem-wide conservation.

## Methods

We compiled count data (1994–2010) from 3 long-term monitoring programs of wintering birds in the Salish Sea: aerial surveys by Washington State's Department of Fish and Wildlife's (WDFW) (Nysewander et al. 2005), British Columbia Coastal Waterbird Surveys (BCCWS) (Crewe et al. 2012), and Audubon Christmas Bird Counts (CBC) (Butcher 1990) (Fig.1). Aerial surveys were conducted using 50 m strip transects on each side of a seaplane traveling at 148–167 km/h at an altitude of about 65 m. Although some birds flush in response to low flying planes and not all species are easily detected, aerial surveys provide extensive coverage of large and inaccessible areas of Puget Sound. The WDFW's aerial surveys have been remarkably consistent because the same crew has been doing them with the same seaplane for the entire 17-year period. The BCCWS involved monthly shore-based counts conducted 2 h before high tide within predefined spatially explicit boundaries by skilled volunteer observers. These surveys extensively covered the Strait of Georgia, but coverage has sometimes been uneven in inaccessible and unpopulated areas. In the CBC surveys counts were conducted during a 24 h midnight-to-midnight calendar day within 2 weeks of 24 December by volunteers with different abilities and using a variety of survey methods. Although yearly differences in methodology are possible, CBC surveys provide valuable long-term data that covers both U.S. and Canadian waters.

After excluding landbirds, we identified 148 marine bird taxa in the survey data. As for most biological surveys covering large temporal and spatial scales, a few species comprised the majority of the observed individuals (Preston 1948); 95% of all species accounts corresponded to only one-fourth of the 148 marine bird taxa. Because our aim was to focus on species that are persistent, abundant, and biologically associated with the Salish Sea, we excluded rare species (those with an average species composition <0.05%) from our analyses. The resulting data set included an ecologically and phylogenetically diverse suite of 39 taxa that we considered the core wintering marine bird community of the Salish Sea (Table1).

**Table 1:** Core taxa of the Salish Sea (Pacific Northwest of North America) wintering marine bird community

*Order, family, species*	*Common name*	*Abbreviation*	*Decline (%)*	*Increase (%)*
Anseriformes			10.9 (67 of 615)	8.9 (55 of 615)
Anatidae			41.8 (28 of 67)	34.3 (23 of 67)
**Dabbling ducks and geese**				
*Anas acuta*	Northern Pintail	NOPI	–	7.5 (5 of 55)
*Anas americana*	American Wigeon	AMWI	–	3 (2 of 55)
*Anas crecca*	Green-winged teal	GWTE	–	3 (2 of 55)
*Anas platyrhynchos*	Mallard	MALL	–	3 (2 of 55)
*Branta bernicla*	Brant	BRAN	–	6 (4 of 55)
*Branta canadensis*	Canada Goose	CAGO	–	3 (2 of 55)
**Diving ducks**			20.9 (14 of 67)	5.5 (3 of 55)
*Aythya.sp*	Scaups	SCAUPS	3 (2 of 67)	–
*Bucephala albeola*	Bufflehead	BUFF	1.5 (1 of 67)	1.5 (1 of 55)
*Bucephala.sp*	Goldeneyes	GOLDENYES	–	–
*Clangula hyemalis*	Long-tailed Duck	LTDU	–	–
*Histrionicus histrionicus*	Harlequin Duck	HARD	–	–
*Lophodytes cucullatus*	Hooded Merganser	HOME	–	–
*Melanitta.sp*	Scoters (Black, Surf and White-winged)	SCOTERS	9 (6 of 67)	–
*Mergus.sp*	Mergansers (Common and Red-Breasted)	MERGANSERS	1.5 (1 of 67)	3 (2 of 55)
*Oxyura jamaicensis*	Ruddy Duck	RUDU	6 (4 of 67)	–
Charadriiformes				
Alcidae			41.8 (28 of 67)	7.5 (5 of 55)
*Brachyramphus marmoratus*	Marbled Murrelet	MAMU	9 (6 of 67)	3 (2 of 55)
*Cepphus columba*	Pigeon Guillemot	PIGU	–	4.5 (3 of 55)
*Cerorhinca monocerata*	Rhinoceros Auklet	RHAU	9 (6 of 67)	0 (0 of 55)
*Synthliboramphus antiquus*	Ancient Murrelet	ANMU	1.5 (1 of 67)	1.5 (1 of 55)
*Uria aalge*	Common Murre	COMU	22.4 (15 of 67)	0 (0 of 55)
Haematopodidae				
*Haematopus bachmani*	Black Oystercatcher	BLOY	0 (0 of 67)	4.5 (3 of 55)
Laridae			7.5 (5 of 67)	16.4 (11 of 55)
*Larus canus*	Mew Gull	MEGU	–	13.4 (9 of 55)
*Larus glaucescens*	Glaucous-winged Gull	GWGU	1.5 (1 of 67)	3 (2 of 55)
*Larus thayeri*	Thayer's Gull	THGU	1.5 (1 of 67)	–
*Chroicocephalus philadelphia*	Bonaparte's Gull	BOGU	4.5 (3 of 67)	–
Scolopacidae				
*Arenaria melanocephala*	Black Turnstone	BLTU	–	3 (2 of 55)
*Calidris alpina*	Dunlin	DUNL	–	1.5 (1 of 55)
Ciconiformes				
Ardeidae				
*Ardea herodias*	Great Blue Heron	GBHE	–	–
Coraciiformes				
Alcedinidea				
*Megaceryle alcyon*	Belted Kingfisher	BEKI	–	–
Falconiformes				
Accipitridae				
*Haliaeetus leucocephalus*	Bald Eagle	BAEA	–	1.5 (1 of 55)
Gaviiformes				
Gaviidae			20.9 (14 of 67)	–
*Gavia immer*	Common Loon	COLO	1.5 (1 of 67)	–
*Gavia pacifica*	Pacific Loon	PALO	1.5 (1 of 67)	–
*Gavia stellata*	Red-throated Loon	RTLO	3 (2 of 67)	–
Suliformes				
Phalacrocoracidae			–	10.4 (7 of 55)
*Phalacrocorax auritus*	Double-crested Cormorant	DCCO	–	7.5 (5 of 55)
*Phalacrocorax pelagicus*	Pelagic Cormorant	PECO	–	–
*Phalacrocorax penicillatus*	Brandt's Cormorant	BRAC	–	3 (2 of 55)
Podicipediformes				
Podicipedidae			23.9 (16 of 67)	6 (4 of 55)
*Aechmophorus occidentalis*	Western Grebe	WEGR	19.4 (13 of 67)	–
*Podiceps auritus*	Horned Grebe	HOGR	1.5 (1 of 67)	–
*Podiceps grisegena*	Red-necked Grebe	RNGR	3 (2 of 67)	6 (4 of 55)

### Trend Analyses

Using generalized least squares models, we evaluated change over time (1994–2010) in mean annual winter counts (log transformed) for the 39 core taxa in each of the 24 Salish basin-depth habitat combinations described in Supporting Information. These models adjusted for the effect of survey type and accounted for the potential serial correlation of counts from successive survey years by including an autoregressive correlation structure of order 1 (AR1) (Pinheiro & Bates 2000). Because we limited trend assessments to species-basin depth habitat combinations with observations in more than one-fourth of the 17 survey years (i.e., dabbling duck trends, for instance, were not evaluated in deep-water habitats of the Strait of Juan de Fuca because they generally do not occur there), 615 species-basin-depth habitat combinations of the 936 possible were evaluated. Using predicted mean annual counts for species encountered in each basin depth combination and corresponding survey type, we then created a binary variable indicating species-basin-depth combinations with declining trends that were unlikely artifacts of chance (α = 0.10) and were decreasing at rates of >3% per year (>50% over the 17 years) in predicted annual counts for all survey types available in that basin-depth habitat combination.

### Multivariate Logistic Regression

Using the binary outcome (with a 1 indicating decline and 0 otherwise) generated from the trend analyses described above, we assessed what types of diets, behaviors, and habitats increased the likelihood of particular bird species to decline. Specifically, we evaluated the following ecological traits as risk factors associated with declining species: primary foraging method, prey preference, breeding status in the Salish Sea (Table2), and use of particular Salish basin-depth habitats described in Supporting Information. We defined primary foraging strategies according to Neslon (1979) and Graaf et al. (1985) and determined broad categories of prey preference based on the ecology and phylogeny of the prey (see Table2 for specific prey types included in each prey choice category). Published natural history accounts were used to create dichotomous variables that described risk factors for each species (Supporting Information). We then used multivariate logistic regression and likelihood ratio tests to identify the most parsimonious combination of risk factors driving declines. A forward stepwise selection algorithm based on likelihood ratio tests (*P* ≤ 0.10) was used to determine which risk factors were included in the logistic regression model. We used likelihood ratios and the Akaike information criterion to evaluate all biologically plausible second-order interactions among the selected main effects (Burnham & Anderson 2002). A Hosmer and Lemeshow goodness-of-fit statistic, which tests convergence between model-predicted and observed probabilities, gave us an overall model goodness of fit. We estimated odds ratios for each main effect in the final model by maximum-likelihood estimation and calculated their 95% confidence intervals with the profile-likelihood method.

**Table 2:** Ecological traits and dietary specializations evaluated as possible factors associated with risk of undergoing declines among marine birds

**Foraging strategy**	
Diving	Surface diving—pursuing prey while swimming underwater using either wings or feet
Surface seizing	Includes picking up prey from the surface and surface plunging
Dabbling	Submerging head and neck or tipping headfirst into water while searching for food
Intertidal wading	Includes beach probing, rock gleaning, intertidal wading, and ambushing
Scavenging	Takes a variety of items including refuse or carrion
**Dietary specialization**	
Forage fish species	Includes herring, sandlance, smelt anchovies, and other schooling species
Demersal fish species	Includes bottom dwelling fishes such as flounders, sculpins, sticklebacks, and gunnels
Fish roe	Typically herring or salmon roe
Snails	Snails limpets and their kin
Bivalves	Mussels and clams
Crustaceans	Krill, crayfish, and crabs
Mammals or birds	Marine mammals, bird chicks, and eggs
Plant material	Plants, seeds, algae, and vegetation
**Breeding**	
Local versus nonlocal breeders	

### Temporal Changes in Community Structure

We used hierarchical clustering in conjunction with nonmetric multidimensional scaling (NMDS) to test for changes in community structure of Salish Sea marine birds over the 17-year study period. Both of these analyses were based on a triangular matrix of Bray-Curtis dissimilarities computed between mean annual species compositions of every pair of survey years. In the NMDS analysis monotone regression and primary treatments of ties were applied, whereas in the cluster analysis the complete-average linkage (furthest neighbor) algorithm was applied (Borcard et al. 2011). We then used analysis of similarity (ANOSIM) to test the hypothesis that communities clustered within groups of survey years are more similar to each other than to communities clustered in a different group of years. This test generates a *P* value and a test statistic (*R*) that indicates the degree of separation between groups, where *R* = 1 indicates complete separation among clusters and an *R* = 0 no separation. Although ANOSIM is analogous to an analysis of variance, it uses similarity matrices and is philosophically allied with NMDS ordination (Legendre & Legendre 2012). All statistical analyses were conducted using a combination of MATLAB (R2013b; MathWorks Inc., Natick, Massachusetts, USA) and R (3.0.2; The R Foundation for Statistical Computing) software.

## Results

### Spatial and Temporal Congruency among Surveys

Aerial surveys by the WDFW included nearshore and offshore habitats in all of the Salish basins (Supporting Information). The CBC surveys covered nearshore habitats of all basins except in Hood Canal, whereas BCCWS covered coastal and inshore habitats of 3 basins: Strait of Georgia, San Juan Islands, and Strait of Juan de Fuca (Supporting Information). As a result, the only basin-depth habitat combinations where all survey programs overlapped were shallow habitats in the Strait of Georgia, San Juan Islands, and Strait of Juan de Fuca. The WDFW and CBC surveys overlapped in shallow habitats of Admiralty Inlet, Central Puget Sound, South Puget Sound, and Whidbey Island basins. The WDFW surveys and BCCWS overlapped in the Strait of Georgia and San Juan Islands basins (Supporting Information). Marine birds inhabiting nearshore habitats were targeted more by the 2 shore-based surveys, whereas the aerial survey targeted species in nearshore and offshore habitats.

### Prevalence of Undergoing Declines

The hypothesis associating changes in community structure of marine birds with bottom–up or top–down driven changes in prey availability (Ballance et al. 1997; Hyrenbach & Veit 2003; Ainley & Hyrenbach 2010), predicts that declines in population size due to changes in food availability are most extreme in species with higher foraging energy expenditure, namely diving birds with high wing loadings. Our results are consistent with this hypothesis. Of the 615 species-basin-depth habitat combinations we assessed, 67 (11%) exhibited 50% or greater declines in winter counts in all survey types (Table1). Diving species accounted for 93% (62 of 67) of all declines, whereas 7% (5 of 67) of declines occurred in surface foraging species (χ^2^ = 22.64, df = 1, *P* ≤ 0.001). In particular, declines were most prevalent among Alcids. Other diving species such as grebes, diving ducks, and loons also exhibited declines (Table1). Instances of species with increasing trends (same criteria as declines but in opposite direction) were more common for surface foraging species. Of the 55 cases of species with increasing trends, 66% (35 of 55) were surface foragers, whereas 38% (20 of 55) were divers (χ^2^ = 24.85, df = 1, *P* = <0.001). Dabbling ducks, gulls, and geese consisted of more than half of all cases of increasing trends. Among diving species with increasing trends, Double-Crested (*Phalacrocorax auritus*) and Brandt's Cormorants (*Phalacrocorax penicillatus*) comprised one-third of all instances of increasing trends. Finally, prevalence of species undergoing declines was relatively equal among basins and depths (likelihood ratio test for significance in declines for basins, *G* = 7.9, df = 7, *P* = 0.333, and for depth habitats, *G* = 2.5, df = 2, *P* = 0.293). The same was not true for prevalence of species with increasing trends (likelihood ratio test for significance in increasing trends for basins, *G* = 5.57, df = 7, *P* < 001, and depth habitats, *G* = 5.57, df = 7, *P* < 001). Species with increasing wintering trends were more common in Admiralty Inlet than in the Strait of Georgia, whereas among depths most instances of species increasing occurred in shallow water habitats.

### Multivariate Logistic Regression

Ecological traits as risk factors associated with declines in mean annual winter counts of Salish Sea marine birds were strongly associated with foraging strategy, dietary specialization, and local breeding status. Specific traits associated with declines included diving as a primary foraging strategy, diets of forage and demersal fish, and whether species breed locally within the Salish Sea (Table3). A logistic model including these covariates demonstrated good overall fit (Hosmer-Lemeshow goodness of fit, χ^2^ = 3.76, *P* = 0.709). This full model indicated that diving birds wintering in the Salish Sea were approximately 11 times more likely to have undergone declines in their winter counts compared with surface-foraging species, such as dabblers, scavengers, and surface seizing or intertidal foraging birds. Furthermore, bird species feeding on forage fish were approximately 8 times more likely to have undergone declines than species that do not feed on forage fish. In contrast, marine birds that include demersal fish as major prey items were less likely to exhibit declines than bird species that did not include demersal fish as major prey. Specifically, piscivorous marine birds that do not prey on demersal fish were approximately 16 times more likely to undergo declines than species that do prey on demersal fishes. Finally, locally breeding species were less likely to undergo declines in winter counts than those species that do not use the Salish Sea for breeding sites. Alternatively, nonlocal breeders were approximately 3 times more likely to decline than species with local breeding colonies.

**Table 3:** Ecological traits identified by logistic regression as risk factors for undergoing declines >50% in mean annual counts among marine birds in the Salish Sea (Pacific Northwest of North America)

*Ecological trait or risk factor*	*Odds ratio*	*95% CI*	**P**
Foraging strategy			
Diving (yes/no)	11.07	4.6 − 33.2	<0.001
Prey choice			
Forage fish (yes/no)	7.66	3.9 − 15.8	<0.001
Demersal fish (yes/no)	0.06	0.03 − 0.14	<0.001
Breeding			
Locally (yes/no)	0.33	0.15 − 0.69	0.003

**Figure 2:**
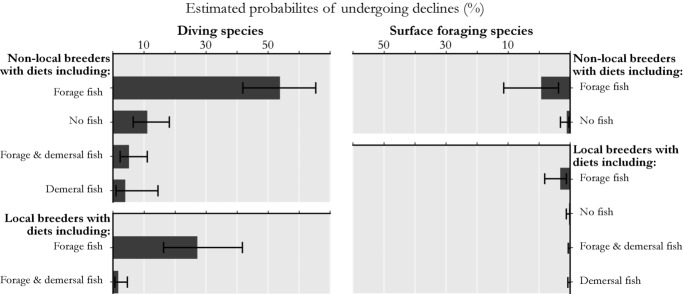
Estimated probabilities of undergoing declines >50% in mean annual counts from 1994 to 2010 for Salish Sea marine birds as a function of ecological traits identified as risk factors by logistic regression (results in Table3). Error bars show 95% confidence intervals.

With the logistic model, we estimated probabilities for wintering birds in particular foraging guilds to decline by over 50% in mean annual counts from 1994 to 2010. In general, probabilities for declining trends were higher for diving birds than for surface foragers and higher for nonlocal versus local breeders (Fig.2). In particular, piscivorous diving birds specializing on forage fish and without local breeding colonies (e.g., Common Murres [*Uria aalge*] and Western Grebes [*Aechmophorus occidentalis*]) had the highest probability of undergoing declines. Tellingly, among nonlocal breeders, piscivorous diving species, including demersal fish (e.g., loon species) and those specializing on demersal fish species (e.g., mergansers), had probabilities of declining that were an order of magnitude lower than diving species specializing on forage fish. In the case of locally breeding piscivorous divers, species including demersal fishes in their diet (e.g., Pigeon Guillemots [*Cepphus Columba*] and Double-Crested Cormorants) had a predicted probability of undergoing regional declines of 2% versus 27% for diving forage fish specialists (e.g., Marbled Murrelets [*Brachyramphus marmoraus*] and Rhinoceros Auklets [*Cerorhinca monocerata*]). Surface foragers with diets relying more on forage fish (e.g., Bonaparte's gulls [*Chroicocephalus philadelphia*]) were also at a disadvantage when compared with other surface foraging species with more generalist diets or those that excluded fish (e.g., dabbling ducks, Dunlins [*Calidris alpina*] and Black Oystercatchers [*Haematopus bachmani*]) (Fig.2).

### Temporal Changes in Community Structure

**Figure 3:**
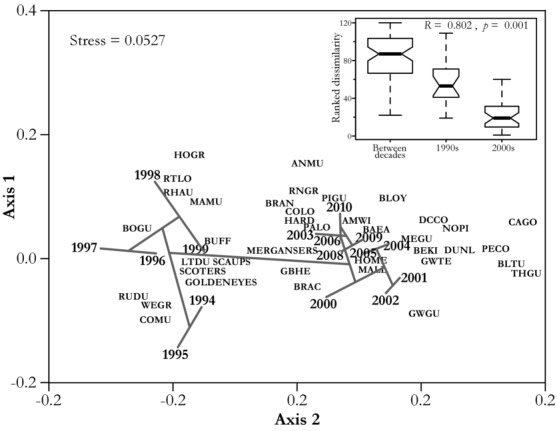
Nonmetric multivariate multidimensional scaling (NMDS) of mean annual winter counts of the 39 core taxa of marine birds in the Salish Sea; stress value of the ordination is shown in the upper left corner. Overlaid on the ordination is a dendrogram of an average linkage cluster tree based on Bray-Curtis dissimilarities among mean annual winter counts of the 39 core taxa. Inset shows results of an analysis of similarity (ANOSIM) test and ranked community structure dissimilarities of pairwise comparisons between survey years grouped in the 1990s and 2000s and within each decade. Boxes represent the median and interquartile range (IQR), and whiskers extend to the most extreme dissimilarities up to 1.5 times the IQR. See Table1 for definitions of species abbreviations.

Ordination and cluster analyses indicated changes in structure of the Salish Sea marine bird community from 1994 to 2010 (Fig.3). With a convergent solution attained in 2 random starts and a minimum stress value of 0.053, NMDS showed a gradient in community composition traced along axis 2. Survey years from the 1990s were grouped together on the left of axis 2, whereas survey years from the 2000s were on the right. Species assemblages associated with these groups were also different. In general, alcids and sea ducks were associated with survey years during the 1990s, whereas nondiving bird species and diving species with diverse diets were associated with survey years in the 2000s. This differentiation of species composition in survey years of different decades is further underlined by the dendrogram projected onto the NMDS ordination in Fig.3. The overlaid average linkage cluster tree based on Bray-Curtis dissimilarities showed a clear separation of the 1994–1999 and 2000–2010 periods. An ANOSIM test that resulted in a high *R* value of 0.802 (*P* = 0.001) further supported the strong separation between community structure during the1990s and 2000s.

## Discussion

Our results reinforce previous spatially restricted research that suggests abundance of wintering marine birds in the Salish Sea has been declining since the mid 1990s. At the larger regional scale, our results indicate that these patterns have been consistent throughout the entire Salish Sea. Additionally, our community-wide trend analyses and subsequent epidemiological synthesis allowed us to identify ecological traits as risk factors that increase the likelihood of species undergoing declines and thus to hypothesize possible mechanisms driving changes in the Salish Sea ecosystem. Species with declining trends were not from random assemblages; instead, they were correlated with specific ecological traits and dietary specializations. In particular, pursuit divers that primarily feed on forage fish and without local breeding colonies were more likely to have declined.

We propose that shifts in the availability and quality of low trophic level prey could explain why diving forage fish specialists were less likely to overwinter in the Salish Sea. This reasoning is founded on evidence of long-term changes in forage fish availability in the Salish Sea and on 2 marine bird ecological concepts. First, regarding forage fish availability, half of all Pacific herring (*Clupea pallasii*) stocks in Puget Sound are either depressed or have such low abundance that recruitment failure is likely or has already occurred (Stick & Lindquist 2009), and in British Columbia the only herring stock within the Salish Sea is experiencing marked declines (Schweigert et al. 2010). Large herring have also proportionally declined in the Salish Sea (Therriault et al. 2009), which may decrease diet quality and calories per catch for diving forage fish specialists (Norris et al. 2007; Schrimpf et al. 2012). And because 93% of Puget Sound's coastline has been altered by removal of shoreline vegetation, dredging, seawalls, and other coastal modifications (Simenstad et al. 2011), the availability of Pacific herring and other forage fish species like surf smelt (*Hypomesus pretiosus*) and Pacific sand lance (*Ammodytes hexapterus*) that spawn in coastal habitats has probably been negatively affected (Shipman et al. 2010).

Second, regarding marine bird ecological concepts, because all species in our study are nonbreeding when overwintering in the Salish Sea, they are unconstrained by having to return to their colonies between foraging bouts to feed chicks and are therefore more likely responding to spatial and temporal variations in the abundance of their prey (Orians & Pearson 1979). Moreover, diving birds typically have high metabolic rates and energetically expensive flight; thus, they need to be close to sufficient prey to meet these high energetic requirements (Pennycuick 1987; Nagy et al. 1999). In contrast, surface foragers can often exploit patchy, widely distributed food sources (Ballance et al. 1997; Hyrenbach & Veit 2003). Therefore, if Salish Sea forage fish availability were to decrease, wintering avifauna more likely to undergo declines should be among diving species—a prediction that is consistent with our results (Table3).

If forage fish availability has decreased in the Salish Sea, concurrent declines in wintering marine birds should be most prevalent in species without local breeding colonies. Although nonlocal breeders use the Salish Sea only for overwintering, locally breeding species rely on the Salish Sea during both breeding and nonbreeding seasons (for breeding sites and winter foraging). And because site fidelity of wide ranging birds is generally stronger to breeding sites than to overwintering sites (Esler 2000), one would expect nonlocal breeders to change winter foraging areas more readily in response to variability in Salish Sea prey availability. For example, in addition to a 52% decline in its North American wintering population from 1975 to 2010, abundance of western grebes in the Salish Sea decreased by about 95% yet increased along the California Coast by over 300% (Wilson et al. 2013). Similar patterns in other migratory species shifting wintering distributions as a result of low Salish Sea forage fish stocks could be reflective of patterns seen in our results (Table3; Figs.2 & 3).

Other top-down and habitat factors may also be contributing to the pattern of declining species we found. For example, nesting sites of Common Murres along the outer coast of British Columbia and Washington—which are likely sources of murres wintering in the Salish Sea—are being influenced by both direct adult and indirect egg mortality (e.g., facilitating crow and gull predation on eggs) due to increasing predation by Bald Eagles (*Haliaeetus leucocephalus*) (Parrish et al. 2001). In the case of diving forage fish specialists that have local breeding colonies, region-wide declines in abundance of Marbled Murrelets—a local breeder, albeit in terrestrial habitats—have coincided with reductions in their nesting habitat (Miller et al. 2012).

Anthropogenic threats like bycatch and oiling, may also explain why diving species were more likely to show declining trends. For instance, diving birds are caught in gill nets either as bycatch (Zydelis et al. 2013) or entangled in derelict fishing gear (Good et al. 2010). Yet, the actual effects these fisheries have on Salish Sea overwintering birds are unclear, in part because after the 1970s various types of fishing gear have been used and commercial salmon (*Oncorhynchus* spp.) fishing efforts, with which marine bird bycatch is most commonly associated, have decreased (Hamel et al. 2009). Fisheries, however, may still affect particular diving species with habits that make them more likely to be entangled. Oils spills are also more likely to affect diving birds because they spend most of their time on the water and typically dive rather than fly when disturbed (Clark 1984). Nevertheless, even though chronic low-level oiling (e.g., small oil spills and bilge dumping) continues to be an issue, incidents of severe oil spills have generally declined (O'Hara et al. 2009).

The strong correlation of declines with diving birds specializing on forage fish supports the hypothesis that declines should be more prevalent in diving species which have higher energy foraging expenditures than surface foragers (Ballance et al. 1997; Hyrenbach & Veit 2003; Ainley & Hyrenbach 2010). Also in support is the apparent change in community structure since 2000 (Fig.3); it suggests that wintering foraging conditions during the 2000s were less favorable for alcids, grebes, and sea ducks, whereas conditions seemed to benefit surface foragers and divers with diverse diets. Other Salish Sea diving predators with more generalist diets also seem to be thriving. Harbor seals (*Phoca vitulina*), for example, which often consume similar forage fish species as diving birds (Lance et al. 2012), have recovered to carrying capacity following major population losses and have presumably stable populations (Jeffries et al. 2003). The eastern Pacific did revert to cool and more productive conditions in late 1998 (Bond et al. 2003; Chavez et al. 2003), as is indicated by the change in the Pacific Decadal Oscillation to a cold phase that mostly persisted through 2010. A change that perhaps is drawing diving forage fish specialist to overwinter in the California Current as a result of poor forage fish prey conditions in the Salish Sea.

Epidemiology seeks to identify risk factors of health-related states or events in populations and thus to inform preventive medicine and support science-based policy. We applied this approach to marine conservation by evaluating what ecological traits made species in a marine bird community more likely to decline over 17 years. Because ecological traits of wildlife result from much longer-term evolutionary pressures, apparent threats revealed through our study are likely due to current changes within the Salish Sea ecosystem. Along with community ecology, epidemiological tools could be applied to other large-scale ecosystems where defining and measuring environmental stressors and their impact on ecosystem health have proven difficult.

## Supporting Information

A figure delineating the major Salish Sea basins and depth habitats used as analysis units (Appendix S1); a table with the core taxa of the Salish Sea marine bird community and their dichotomously categorized foraging strategies, primary choices of prey, and local breeding status (Appendix S2); and time frames of monitoring programs within the basin depth habitat combinations (Appendix S3) are available online. The authors are solely responsible for the content and functionality of these materials. Queries (other than absence of the material) should be directed to the corresponding author.
