# Supplementary material for: Assessing ecological correlates of marine bird declines to inform marine conservation
Source: Conserv Biol. 2014 Sep 5;29(1):154–63. doi: 10.1111/cobi.12378 (PMC4322479; doi:10.1111/cobi.12378)
Supplement: Supplementary file 1 — A figure delineating the major Salish Sea basins and depth habitats used as analysis units (Appendix S1); a table with the core taxa of the Salish Sea marine bird community and their dichotomously categorized foraging strategies, primary choices of prey, and local breeding status (Appendix S2); and time frames of monitoring programs within the basin depth habitat combinations (Appendix S3) are available online. The authors are solely responsible for the content and functionality of these materials. Queries (other than absence of the material) should be directed to the corresponding author. [file cobi0029-0154-sd1.zip › cobi12378-sup-0002-appendixS2.docx]

**Appendix S2** Core taxa of the Salish Sea marine bird community and their dichotomously categorized foraging strategies, primary choices of prey and local breeding status

| Order, Family, Species | | | Common name |  | Primary foraging strategy | | | | |  | Dietary preferences | | | | | | | |  | Breeding |
| --- | --- | --- | --- | --- | --- | --- | --- | --- | --- | --- | --- | --- | --- | --- | --- | --- | --- | --- | --- | --- |
|  |  |  |  |  | diving | surface seizing | dabbling | intertidal foraging | scavenging |  | forage fish | demersal fish | fish roe | mammals &/or birds | snails | mussels | crustaceans | plants |  | includes the Salish Sea |
| Anseriformes | | |  |  |  |  |  |  |  |  |  |  |  |  |  |  |  |  |  |  |
|  | Anatidae | |  |  |  |  |  |  |  |  |  |  |  |  |  |  |  |  |  |  |
|  |  | *Anas acuta* | Northern Pintail |  | no | no | yes*^1, 2^* | yes*^1, 2^* | no |  | no | no | no | no | yes*^2, 3, 4^* | no | yes*^2, 3, 4^* | yes*^2, 3, 4^* |  | no |
|  |  | *Anas americana* | American Wigeon |  | no | no | yes*^1, 2^* | yes*^1, 2^* | no |  | no | no | no | no | no | no | no | yes*^2, 3, 4^* |  | no |
|  |  | *Anas crecca* | Green-Winged teal |  | no | no | yes*^1, 2^* | yes*^1, 2^* | no |  | no | no | no | no | no | no | yes*^2, 3, 4^* | yes*^2, 3, 4^* |  | no |
|  |  | *Anas platyrhynchos* | Mallard |  | no | no | yes*^1, 2^* | yes*^1, 2^* | no |  | no | no | no | no | no | no | no | yes*^2, 3, 4^* |  | yes*^4, 5^* |
|  |  | *Branta bernicla* | Brant |  | no | no | yes*^1, 2^* | yes*^1, 2^* | no |  | no | no | no | no | no | no | no | yes*^2, 3, 4^* |  | no |
|  |  | *Branta canadensis* | Canada Goose |  | no | no | yes*^1, 2^* | no | no |  | no | no | no | no | no | no | no | yes*^2, 4^* |  | yes*^4, 5^* |
|  |  | *Aythya.sp* | Scaup Species |  | yes*^2, 6^* | no | no | no | no |  | no | no | yes*^2, 4, 7^* | no | yes*^2, 4, 7^* | yes*^2, 4, 7^* | yes*^2, 4, 7^* | yes*^2, 4, 7^* |  | no |
|  |  | *Bucephala albeola* | Bufflehead |  | yes*^2, 6^* | no | no | no | no |  | no | no | yes*^2, 4, 8^* | no | yes*^2, 4, 8^* | yes*^2, 4, 8^* | yes*^2, 4, 8^* | no |  | no |
|  |  | *Bucephala.sp* | Goldeneyes |  | yes*^2, 6^* | no | no | no | no |  | no | no | yes*^2, 4, 9^* | no | yes*^2, 4, 9^* | yes*^2, 4, 9^* | yes*^2, 4, 9^* | no |  | no |
|  |  | *Clangula hyemalis* | Long-tailed Duck |  | yes*^2, 6^* | no | no | no | no |  | no | no | yes*^2, 4^* | no | yes*^2, 4, 9^* | yes*^2, 4, 9^* | yes*^2, 4, 9^* | no |  | no |
|  |  | *Histrionicus histrionicus* | Harlequin Duck |  | yes*^2, 6^* | no | no | no | no |  | no | no | yes*^4, 10, 11^* | no | yes*^4, 10, 11^* | yes*^4, 10, 11^* | yes*^4, 10, 11^* | no |  | no |
|  |  | *Lophodytes cucullatus* | Hooded Merganser |  | yes*^2, 6^* | no | no | no | no |  | no | yes*^2, 4^* | no | no | no | no | yes*^2, 4^* | no |  | no |
|  |  | *Melanitta.sp* | Scoter species |  | yes*^2, 6^* | no | no | no | no |  | no | no | yes*^4, 8, 12^* | no | yes*^4, 8^* | yes*^4, 8^* | yes*^4, 8^* | no |  | no |
|  |  | *Mergus.sp* | Merganser species |  | yes*^2, 6^* | no | no | no | no |  | no | yes*^2, 4, 13^* | yes*^2, 4, 13^* | no |  |  | yes*^2, 4, 13^* | no |  | no |
|  |  | *Oxyura jamaicensis* | Ruddy Duck |  | yes*^2, 6^* | no | no | no | no |  | no | no | no | no | yes*^2, 4, 8^* | yes*^2, 4, 8^* | yes*^2, 4, 8^* | yes*^2, 4, 8^* |  | no |
| Charadriiformes | | |  |  |  |  |  |  |  |  |  |  |  |  |  |  |  |  |  |  |
|  | Alcidae | |  |  |  |  |  |  |  |  |  |  |  |  |  |  |  |  |  |  |
|  |  | *Brachyramphus marmoratus* | Marbled Murrelet |  | yes*^2, 14^* | no | no | no | no |  | yes*^2, 14, 15^* | no | no | no | no | no | yes*^2, 14, 15^* | no |  | yes*^4, 5^* |
|  |  | *Cepphus columba* | Pigeon Guillemot |  | yes*^2, 14^* | no | no | no | no |  | yes*^2, 14, 15^* | yes*^2, 14, 15^* | no | no | no | no | yes*^2, 14, 15^* | no |  | yes*^4, 5^* |
|  |  | *Cerorhinca monocerata* | Rhinoceros Auklet |  | yes*^2, 14^* | no | no | no | no |  | yes*^2, 14, 15^* | no | no | no | no | no | no | no |  | yes*^4, 5^* |
|  |  | *Synthliboramphus antiquus* | Ancient Murrelet |  | yes*^2, 14^* | no | no | no | no |  | yes*^2, 14, 15^* | no | no | no | no | no | yes*^2, 14, 15^* | no |  | no |
|  |  | *Uria aalge* | Common Murre |  | yes*^2, 14^* | no | no | no | no |  | yes*^2, 14, 15^* | no | no | no | no | no | no | no |  | no |
|  | Haematopodidae | |  |  |  |  |  |  |  |  |  |  |  |  |  |  |  |  |  |  |
|  |  | *Haematopus bachmani* | Black Oystercatcher |  | no | no | no | yes*^2, 16^* | no |  | no | no | no | no | yes*^2, 16^* | yes*^2, 16^* | yes*^2, 16^* | no |  | yes*^4, 5^* |
|  | Laridae | |  |  |  |  |  |  |  |  |  |  |  |  |  |  |  |  |  |  |
|  |  | *Larus canus* | Mew Gull |  | no | yes*^2, 17^* | no | yes*^2, 17^* | yes*^2, 17^* |  | yes*^4, 17^* | no | yes*^4, 17^* | yes*^4, 17, 18^* | yes*^4, 17, 18^* | yes*^4, 17, 18^* | yes*^4, 17, 18^* | no |  | yes*^4, 5^* |
|  |  | *Larus glaucescens* | Glaucous-winged Gull |  | no | yes*^2, 17^* | no | yes*^2, 17^* | yes*^2, 17^* |  | yes*^4, 17^* | no | yes*^4, 17^* | yes*^4, 17, 18^* | yes*^4, 17, 18^* | yes*^4, 17, 18^* | yes*^4, 17, 18^* | no |  | yes*^4, 5^* |
|  |  | *Larus thayeri* | Thayer's Gull |  | no | yes*^2, 17^* | no | yes*^2, 17^* | yes*^2, 17^* |  | yes*^4, 17^* | no | yes*^4, 17^* | yes*^4, 17, 18^* | yes*^4, 17, 18^* | yes*^4, 17, 18^* | yes*^4, 17, 18^* | no |  | no |
|  |  | *Chroicocephalus philadelphia* | Bonaparte's Gull |  | no | yes*^2, 17^* | no | yes*^2, 17^* | no |  | yes*^4, 17^* | no | yes*^4, 17^* | no | no | no | yes*^4, 17, 18^* | no |  | no |
|  | Scolopacidae | |  |  |  |  |  |  |  |  |  |  |  |  |  |  |  |  |  |  |
|  |  | *Arenaria melanocephala* | Black turnstone |  | no | no | no | yes*^2, 4^* | no |  | no | no | yes*^4^* | no | yes*^4^* | yes*^4^* | yes*^4^* | no |  | no |
|  |  | *Calidris alpina* | Dunlin |  | no | no | no | yes*^2, 4^* | no |  | no | no | no | no | yes*^4^* | yes*^4^* | yes*^4^* | no |  | no |
| Ciconiiformes | | |  |  |  |  |  |  |  |  |  |  |  |  |  |  |  |  |  |  |
|  | Ardeidae | |  |  |  |  |  |  |  |  |  |  |  |  |  |  |  |  |  |  |
|  |  | *Ardea herodias* | Great blue heron |  | no | yes*^2, 4^* | no | yes*^2, 4^* | no |  | no | yes*^2, 4^* | no | yes*^2, 4^* | no | no | no | no |  | yes*^4, 5^* |
| Coraciiformes | | |  |  |  |  |  |  |  |  |  |  |  |  |  |  |  |  |  |  |
|  | Alcedinidea | |  |  |  |  |  |  |  |  |  |  |  |  |  |  |  |  |  |  |
|  |  | *Megaceryle alcyon* | Belted kingfisher |  | no | yes*^2, 4^* | no | no | no |  | yes*^2, 4^* | no | no | no | no | no | no | no |  | yes*^4, 5^* |
| Falconiformes | | |  |  |  |  |  |  |  |  |  |  |  |  |  |  |  |  |  |  |
|  | Accipitridae | |  |  |  |  |  |  |  |  |  |  |  |  |  |  |  |  |  |  |
|  |  | *Haliaeetus leucocephalus* | Bald eagle |  | no | yes*^2, 19^* | no | no | yes*^2, 19^* |  | yes*^4, 20^* | yes*^4, 20^* | no | yes*^4, 20^* | no | no | no | no |  | yes*^4, 5^* |
| Gaviiformes | | |  |  |  |  |  |  |  |  |  |  |  |  |  |  |  |  |  |  |
|  | Gaviidae | |  |  |  |  |  |  |  |  |  |  |  |  |  |  |  |  |  |  |
|  |  | *Gavia immer* | Common Loon |  | yes*^2, 21^* | no | no | no | no |  | yes*^4, 22^* | yes*^4, 22^* | no | no | no | no | no | no |  | no |
|  |  | *Gavia pacifica* | Pacific Loon |  | yes*^2, 21^* | no | no | no | no |  | yes*^4, 22^* | yes*^4, 22^* | no | no | no | no | no | no |  | no |
|  |  | *Gavia stellata* | Red-throated Loon |  | yes*^2, 21^* | no | no | no | no |  | yes*^4, 22^* | yes*^4, 22^* | no | no | no | no | no | no |  | no |
| Suliformes | | |  |  |  |  |  |  |  |  |  |  |  |  |  |  |  |  |  |  |
|  | Phalacrocoracidae | |  |  |  |  |  |  |  |  |  |  |  |  |  |  |  |  |  |  |
|  |  | *Phalacrocorax auritus* | Double-crested Cormorant |  | yes*^2, 21^* | no | no | no | no |  | yes*^4, 22^* | yes*^4, 22^* | no | no | no | no | no | no |  | yes*^4, 5^* |
|  |  | *Phalacrocorax pelagicus* | Pelagic Cormorant |  | yes*^2, 21^* | no | no | no | no |  | yes*^4, 22^* | yes*^4, 22^* | no | no | no | no | yes*^4, 22^* | no |  | yes*^4, 5^* |
|  |  | *Phalacrocorax penicillatus* | Br&t’s Cormorant |  | yes*^2, 21^* | no | no | no | no |  | yes*^4, 22^* | yes*^4, 22^* | no | no | no | no | no | no |  | no |
| Podicipediformes | | |  |  |  |  |  |  |  |  |  |  |  |  |  |  |  |  |  |  |
|  | Podicipedidae | |  |  |  |  |  |  |  |  |  |  |  |  |  |  |  |  |  |  |
|  |  | *Aechmophorus occidentalis* | Western Grebe |  | yes*^2, 21^* | no | no | no | no |  | yes*^4, 22^* | no | no | no | no | no | no | no |  | no |
|  |  | *Podiceps auritus* | Horned Grebe |  | yes*^2, 21^* | no | no | no | no |  | no | yes*^4, 22^* | no | no | no | no | yes*^4, 22^* | no |  | no |
|  |  | *Podiceps grisegena* | Red-necked Grebe |  | yes*^2, 21^* | no | no | no | no |  | yes*^4, 22^* | yes*^4, 22^* | no | no | no | no | no | no |  | no |

**References**: ^1^Baldassarre & Bolen 2006, ^2^Graaf, et al. 1985, ^3^Baldwin & Lovvorn 1994, ^4^Angell & Balcomb 1982, ^5^Wahl, et al. 2005, ^6^Raikow 1973, ^7^Munro 1941, ^8^Cottam 1935, ^9^Vermeer 1982, ^10^Gaines & Fitzner 1987, ^11^Vermeer 1983, ^12^Bayer 1980, ^13^Munro & Clemens 1939, ^14^Gaston & Jones 1998, ^15^Vermeer, et al. 1987, ^16^Hartwick 1976, ^17^Burger 1988, ^18^Tangren 1982, ^19^Knight & Knight 1983, ^20^Knight, et al. 1990, ^21^Ballance, et al. 2001, ^22^Ainley & Sanger 1979

**Literature Cited**

Ainley, D. G., and G. A. Sanger. 1979. Trophic relations of seabirds in the northeastern Pacific Ocean and Bering Sea. Pages 95–122 in J. C. Bartonek, and D. N. Nettleship, editors. Conservation of marine birds of northern North America. United States Fish and Wildlife Service-Wildlife Research Report 11, Washington, D.C.

Angell, T., and K. C. Balcomb 1982. Marine birds and mammals of Puget Sound. Washington Sea Grant Program, University of Washington Press.

Baldassarre, G. A., and E. G. Bolen 2006. Waterfolw ecology and management. Krieger Publishing Company.

Baldwin, J., and J. Lovvorn. 1994. Habitats and tidal accessibility of the marine foods of dabbling ducks and brant in Boundary Bay, British Columbia. Marine Biology **120**:627–638.

Ballance, L. T., D. G. Ainley, and G. L. Hunt Jr. 2001. Seabird Foraging Ecology. Pages 2636–2644 in S. A. Thorpe, and K. K. Turekian, editors. Encyclopedia of Ocean Sciences. Academic Press, London.

Bayer, R. D. 1980. Birds feeding on herring eggs at the Yaquina Estuary, Oregon. The Condor **82**:193–198.

Burger, J. 1988. Foraging behavior in gulls: differences in method, prey, and habitat. Colonial Waterbirds **11**:9–23.

Cottam, C. 1935. Food habits of North American diving ducks. United States Department of Agriculture, Wahington, D.C.

Gaines, W. L., and R. E. Fitzner. 1987. Winter diet of the Harlequin diet at Sequim Bay, Puget Sound, Washington. Northwest Science **61**:213–215.

Gaston, A. J., and I. L. Jones 1998. The Auks *Alcidea*. Oxford University Press, New York.

Graaf, R. M., N. G. Tilghman, and S. H. Anderson. 1985. Foraging guilds of North American birds. Environmental Management **9**:493–536.

Hartwick, E. B. 1976. Foraging strategy of the black oyster catcher (*Haematopus bachmani* *Audubon*). Canadian Journal of Zoology **54**:142–155.

Knight, R. L., P. J. Randolph, G. T. Allen, L. S. Young, and R. J. Wigen. 1990. Diets of nesting bald eagles, Haliaeetus leucocephalus in western Washington. The Canadian field-Naturalist **104**:545–551.

Knight, S. K., and R. L. Knight. 1983. Aspects of food finding by wintering bald eagles. The Auk **100**:477–484.

Munro, J. A. 1941. Studies of waterfowl in British Columbia: Greater Scaup duck, Lesser Scaup duck. Canadian Journal of Zoology **19**:113–138.

Munro, J. A., and W. A. Clemens. 1939. The food and feeding habits of the red–breasted merganser in British Columbia. The Journal of Wildlife Management **3**:46–53.

Raikow, R. J. 1973. Locomotor Mechanisms in North American ducks. The Wilson Bulletin **85**:295–307.

Tangren, G. V. 1982. Feeding behavior of crows and gulls on a Puget Sound beach. Western Birds **12**:1–12.

Vermeer, K. 1982. Food and distribution of the three *Bucephala* species in British Columbia waters. Wildfowl **33**.

Vermeer, K. 1983. Diet of the Harlequin Duck in the Strait of Georgia, British Columbia. The Murrelet **64**:54–57.

Vermeer, K., S. G. Sealy, and G. Sanger, A. 1987. Feeding ecology of the Alcidae in the eastern North Pacific in J. P. Croxall, editor. Seabirds: feeding ecology and role in marine ecosystems. Cambridge University Press.

Wahl, T. R., B. Tweit, and S. G. Mlodinow 2005. Birds of Washington. Oregon State University Press, Corvallis, Oregon.
