# Supplementary figures and images for: Assessing ecological correlates of marine bird declines to inform marine conservation
Source: Conserv Biol. 2014 Sep 5;29(1):154–63. doi: 10.1111/cobi.12378 (PMC4322479; doi:10.1111/cobi.12378)

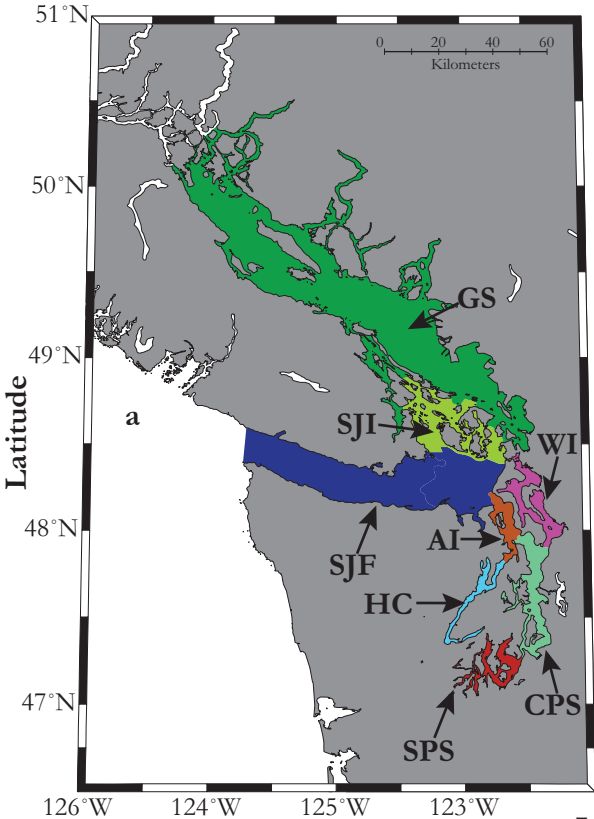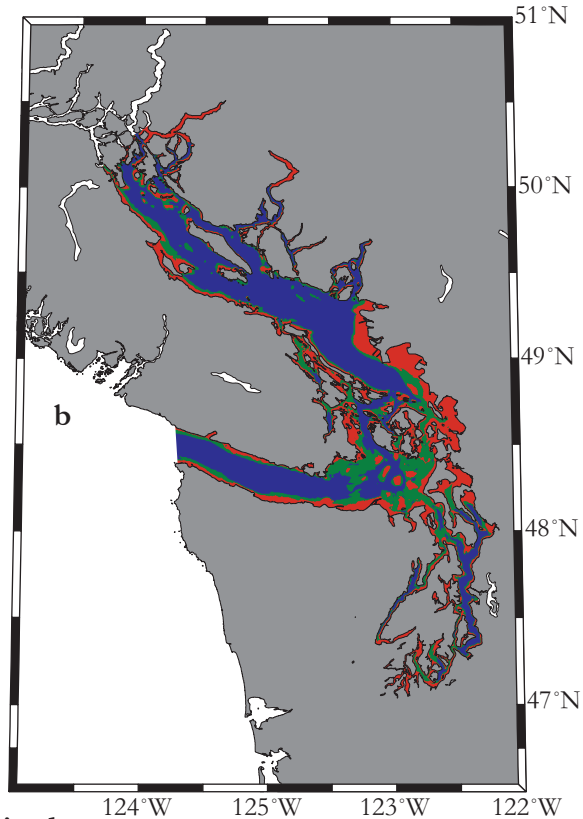

Supplement: Supplementary file 1 — A figure delineating the major Salish Sea basins and depth habitats used as analysis units (Appendix S1); a table with the core taxa of the Salish Sea marine bird community and their dichotomously categorized foraging strategies, primary choices of prey, and local breeding status (Appendix S2); and time frames of monitoring programs within the basin depth habitat combinations (Appendix S3) are available online. The authors are solely responsible for the content and functionality of these materials. Queries (other than absence of the material) should be directed to the corresponding author. [file cobi0029-0154-sd1.zip › cobi12378-sup-0001-appendixS1.pdf]
